# Brain asymmetry is encoded at the level of axon terminal morphology

**DOI:** 10.1186/1749-8104-3-9

**Published:** 2008-03-31

**Authors:** Isaac H Bianco, Matthias Carl, Claire Russell, Jonathan DW Clarke, Stephen W Wilson

**Affiliations:** 1 Department of Cell and Developmental Biology, UCL, Gower St, London WC1E 6BT, UK; 2 Department of Veterinary Basic Sciences, Royal Veterinary College, Royal College St, London NW1 0TU, UK

## Abstract

**Background:**

Functional lateralization is a conserved feature of the central nervous system (CNS). However, underlying left-right asymmetries within neural circuitry and the mechanisms by which they develop are poorly described.

**Results:**

In this study, we use focal electroporation to examine the morphology and connectivity of individual neurons of the lateralized habenular nuclei. Habenular projection neurons on both sides of the brain share a stereotypical unipolar morphology and elaborate remarkable spiraling terminal arbors in their target interpeduncular nucleus, a morphology unlike that of any other class of neuron described to date. There are two quite distinct sub-types of axon arbor that differ both in branching morphology and in their localization within the target nucleus. Critically, both arbor morphologies are elaborated by both left and right-sided neurons, but at greatly differing frequencies. We show that these differences in cell type composition account for the gross connectional asymmetry displayed by the left and right habenulae. Analysis of the morphology and projections of individual post-synaptic neurons suggests that the target nucleus has the capacity to either integrate left and right inputs or to handle them independently, potentially relaying information from the left and right habenulae within distinct downstream pathways, thus preserving left-right coding. Furthermore, we find that signaling from the unilateral, left-sided parapineal nucleus is necessary for both left and right axons to develop arbors with appropriate morphology and targeting. However, following parapineal ablation, left and right habenular neurons continue to elaborate arbors with distinct, lateralized morphologies.

**Conclusion:**

By taking the analysis of asymmetric neural circuitry to the level of single cells, we have resolved left-right differences in circuit microarchitecture and show that lateralization can be recognized at the level of the morphology and connectivity of single projection neuron axons. Crucially, the same circuitry components are specified on both sides of the brain, but differences in the ratios of different neuronal sub-types results in a lateralized neural architecture and gross connectional asymmetry. Although signaling from the parapineal is essential for the development of normal lateralization, additional factors clearly act during development to confer left-right identity upon neurons in this highly conserved circuit.

## Background

The left (L) and right (R) sides of the central nervous system (CNS) display functional asymmetries throughout the animal kingdom [1-3]. There is evidence that functional lateralization increases cognitive performance as well as having important consequences for social behaviors within populations and for interactions between species [4]. At the neuroanatomical level, asymmetries have been described in the shape and size of comparible regions on the R and L sides of the brain, in subnuclear and cytoarchitectonic organization of particular nuclei, as well as at the level of neurotransmitter expression and gross connectivity patterns [5,6]. However, little is currently known about lateralization at the level of individual neurons with respect to the configuration of functional circuits that is expected to underlie LR differences in neural processing. A probable reason for this is that such asymmetries are likely to be very subtle, encoded at the level of dendrite [7] or axon morphology and/or connectivity and/or at the level of synaptic organization [8].

**Figure 1:**
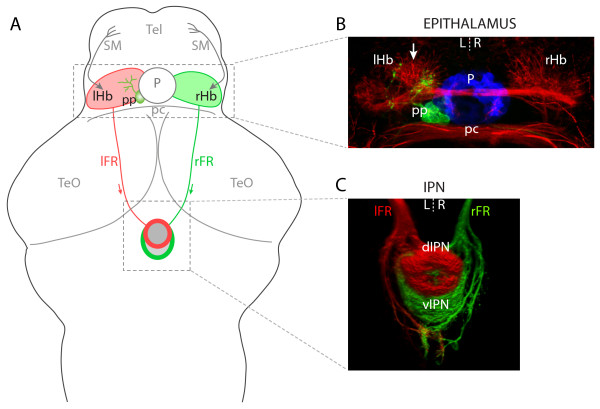
Left-right asymmetries in the telencephalon-habenula-IPN pathway. **(a) **Schematic of a 4 dpf larval zebrafish brain. The bilaterally paired epithalamic habenular (Hb) nuclei receive afferent inputs from nuclei in the telencephalon (Tel) via the stria medullaris (SM; grey arrows; asymmetric innervation of the R habenula [20] is not indicated) and diencephalon (not shown). Habenular neurons send efferent projections via the fasciculi retroflexus (FR) to the interpeduncular nucleus (IPN) in the ventral midbrain. L- and R-sided habenular axons are segregated along the DV axis of the IPN wherein the L habenula predominantly innervates the dIPN and makes less substantial projections to the vIPN whereas the vast majority of R-sided axons terminate in the vIPN. In addition to the habenulae, the epithalamus contains the pineal complex, comprising the photoreceptive pineal (P) and parapineal (pp) nuclei. The parapineal is located on the L side of the dorsal midline and projects efferent axons that exclusively innervate the L habenula. **(b) **Neuroanatomical asymmetries in the epithalamus. Anti-acetylated tubulin immunostaining (red) reveals the L habenula displays a greater density of neuropil, especially in the dorso-medial aspect of the nucleus (arrow). The pineal (blue) and parapineal (green) nuclei are visualized by expression of GFP in a Tg(*foxD3:GFP*) transgenic larva. Parapineal efferent axons predominantly terminate in the asymmetric medial neuropil of the L habenula. **(c) **Three-dimensional reconstruction of habenular axon terminals in the ventral midbrain, labeled using lipophilic carbocyanine dyes applied to the habenulae. L-sided axons were labeled with DiD (red) and R-sided axons with DiI (green). The dIPN is almost exclusively innervated by L-sided axons, whereas the ventral target receives a majority of R-sided inputs. All panels show dorsal views, anterior top. pc, posterior commissure; TeO, optic tectum.

The telencephalo-habenulo-interpeduncular system is an emerging model for studying brain asymmetries and their development [6,9]. The bilateral habenular nuclei, located in the diencephalic epithalamus, are part of this evolutionarily conserved conduction system. These nuclei receive afferent inputs from the basal telencephalon and diencephalon and project efferent axons through the fasciculus retroflexus (FR) to an unpaired midline target, the interpeduncular nucleus (IPN) of the ventral midbrain [10]. The epithalamus also contains the pineal complex, which in zebrafish comprises two photoreceptive nuclei, the pineal and parapineal (Figure 1).

Epithalamic asymmetries are present throughout the vertebrate lineage [11] and are especially conspicuous in anamniotes. In larval zebrafish, the parapineal organ has a bilateral origin, but parapineal precursors migrate from the antero-dorsal epithalamus to the L side of the dorsal midline and contemporaneously extend efferent axons that exclusively innervate the L habenula [12,13]. The L and R habenulae differ in the extent and organization of their neuropil and in the expression of various genes [12-15]. Furthermore, asymmetry extends to the efferent connectivity between the habenulae and the IPN. Habenular axons innervate the IPN in a laterotopic manner wherein L and R terminals are segregated along the dorso-ventral (DV) axis of the target [15,16].

In this study, we have used focal electroporation to examine individual pre- and post-synaptic neurons within the habenulo-interpeduncular tract, enabling us to resolve LR asymmetry in circuit microarchitecture. Our results uncover a fundamental aspect of CNS lateralization that serves to differentiate functional circuitry on the L and R sides of the brain. Additionally, the study extents current understanding of how brain asymmetry develops.

## Results

### L and R habenulae are coherent nuclei populated by unipolar projection neurons

**Figure 2:**
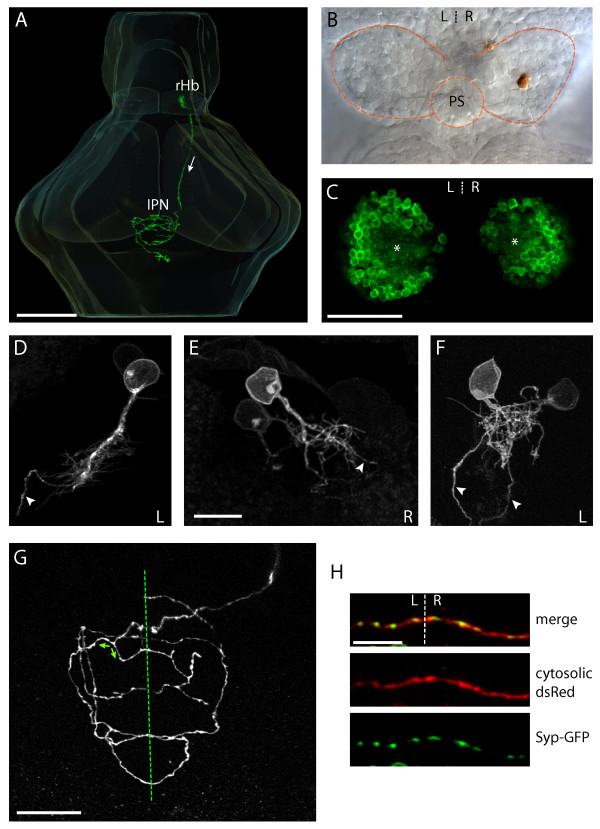
Habenular neurons have a stereotypical unipolar morphology and their axons terminate in spiral-shaped arbors that display multiple midline crossing. **(a) **Three-dimensional reconstruction showing a R habenular (rHb) projection neuron in an intact 4 dpf larval zebrafish brain. Arrow indicates direction of axonal projection within the FR, from the rHb to the IPN. **(b) **A single R habenular neuron labeled by focal electroporation and visualized by anti-GFP immunostaining (brown). The image shows the dorsal diencephalon of a dissected brain of a 4 dpf larva. Dotted lines show the borders of the habenulae and the position of the pineal stalk (PS). **(c) **Single-depth confocal section through the habenulae of a 4 dpf Tg(ET16:GFP) transgenic larva in which a subset of habenular neurons express GFP. In each nucleus the neuronal somata are arranged as ovoid shells surrounding a central neuropil domain (asterisks). It is in this domain that neurons elaborate their dendritic arbors. **(d-f) **Examples of the somata, dendritic arborizations and proximal axons of habenular neurons labeled by focal electroporation of membrane-tethered GFP. (d, e) Neurons with long processes that give rise to a dendritic tree and axon. (f) Two neurons with intertwined dendritic arbors close to the cell body. In (d-f) an arrowhead marks the proximal axon, and the laterality (left (L) or right (R)) of the habenular neuron(s) is indicated bottom right. **(g) **Confocal *z*-projection within the IPN showing a single axonal arbor elaborated by a R habenular projection neuron labeled by focal electroporation. The arbor crosses the ventral midline (dotted line) multiple times. Branches can also reverse direction such that they encircle the IPN in opposite senses (green arrows show examples). **(h) **High-magnification images of a section of a habenular axon arbor crossing the ventral midline (dotted line). The neuron was electroporated with a construct driving expression of cytosolic DsRed (red, middle panel) and a Syp-GFP fusion protein (green, lower panel). Syp-GFP puncta are present on both sides of the midline and co-localise with axonal variscocities. Scale bars: (a) 100 μm; (c) 50 μm; (e, h) 10 μm; (g) 20 μm.

The larval habenular nuclei are discrete, well de-limited and coherent groups of neurons on L and R sides of the brain (Figure 2b,c). They are asymmetric and levels of expression of several genes vary within each nucleus and between nuclei on L and R. However, as individual habenular neurons have not been studied, the extent of neuronal diversity within, and between, L and R nuclei is not known. To investigate this issue, we used focal electroporation to express membrane-tethered green fluorescent protein (GFP) in individual neurons or small groups of habenular neurons, enabling visualization of the entire morphology and axonal projections of these cells in the intact brain. This novel and powerful approach enables detailed comparison between neurons at the level of the soma, dendrites, axons and terminals. Given the small size of the habenulae at the stage of labeling (48–72 hours post-fertilization (hpf)), no attempt was made to target different positions within the nucleus and so the position of labeled neurons was essentially randomized. High-resolution imaging was performed on 83 individual habenular neurons (37 L-sided and 46 R-sided).

By far the majority of cells on both L and R were habenulo-interpeduncular projection neurons that extended axons in the FR and innervated the IPN (95.2%). In four cases, neurons projected more caudal than the IPN, most likely to the serotonergic raphé nucleus in the anterior hindbrain (4.8%; Additional file 1). The axons of these neurons also coursed in the FR, but passed ipsilaterally around the ventral IPN (vIPN), before converging medially, crossing the ventral midline and finally terminating close to it. From here on we focus solely upon the majority population of habenular neurons with axons that terminate in the IPN.

Both L and R habenular nuclei have a central domain of dense neuropil surrounded by an ovoid shell of projection neurons (Figure 2b,c) that elaborate dendrites within the neuropil. All labeled projection neurons, wherever they were located within the shell on either L or R sides, have a unipolar somal morphology (Figure 2d–f). In all cases, neuronal somata extend a single process directed towards the central neuropil where their dendritic trees are elaborated. While all neurons showed this same basic dendritic structure, they varied with respect to branch complexity. We did not determine if this relates to differences in neuronal location or correlates with molecular differences between neurons. In all cases, the axon emerges from one branch of the dendritic arbor and extends, unbranched, within the FR towards the IPN. The neurite arising from the soma and from which both dendrites and axon emerge is variable in length (Figure 2d,e versus 2f), likely reflecting the proximity of the soma to the central neuropil region.

Although there is clearly diversity among habenular projection neurons (see Discussion), these results show that all neurons in both L and R habenulae have in common a basic, stereotypical unipolar morphology.

### Axons of habenular projection neurons cross the midline multiple times and form both ipsilateral and contralateral synaptic contacts

Bulk labeling of habenular efferent axons using lipophilic dyes has shown that both L and R nuclei project to both L and R sides of the target IPN nucleus [16]. The two most parsimonious explanations for this are either that each habenula contains discrete ipsilaterally and contralaterally projecting neurons or that individual neurons have axons that bifurcate and terminate on both sides of the midline. To resolve this issue, we reconstructed the entire terminal morphologies of individual L- and R-sided habenular projection neurons.

The most striking feature of both L- and R-sided axons is that they cross the ventral midline of the CNS multiple times, forming profusely branched 'spirals' of neurites, unlike any other axon type we are aware of (Figure 2g). One hypothesis we considered was that L- and R-sided neurons might 'spiral' in opposite senses (clockwise versus counter-clockwise); however, this is not the case and indeed, branches from individual neurons frequently reverse sense within the arbor.

**Figure 3:**
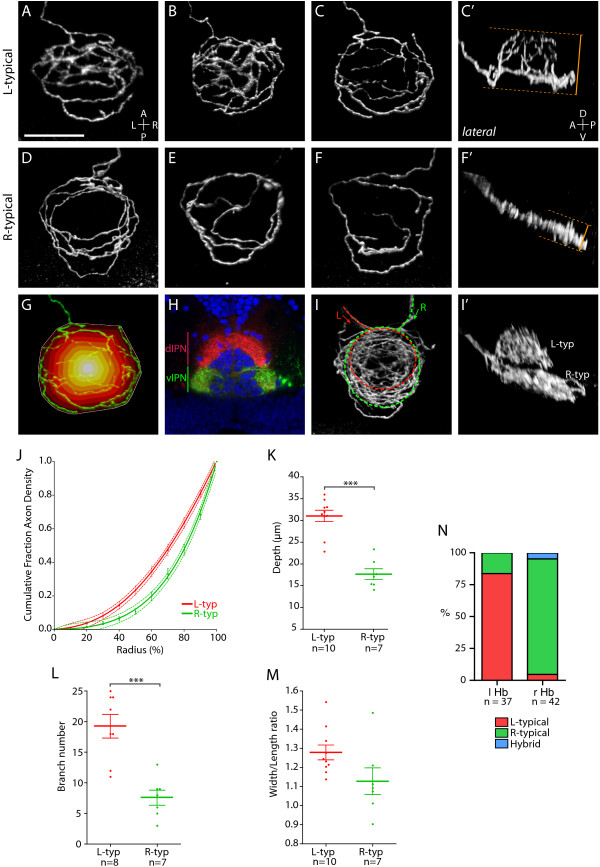
Two distinct axon arbor morphologies formed by habenular projection neurons. **(a-g, i) **Dorsal, **(c', f', i') **lateral and **(h) **transverse confocal images of the IPN region in 4 dpf larvae. In all panels habenular axons were labeled by electroporation except for (h) where lipophilic dye tracing was used to visualize axon terminals. Panels show three-dimensional reconstructions except (h), which is a single-depth confocal image. (a-c') Three examples of L-typical (L-typ) arbors. These arbors are shaped like a domed crown and arborize over a considerable DV extent (compare dorsal (c) and lateral (c') views of an example L-typical arbor). See also Additional file 2. (d-f') Examples of R-typical (R-typ) arbors, which are considerably flatter. See also Additional file 3. Panel (d) shows two R-typical arbors formed by two R habenular neurons. In (c', f') orange dotted lines and bars indicate how DV extent was measured for L-typical and R-typical arbors (see Materials and methods). (g) Schematic to illustrate how the radial distribution of axon density was measured. The perimeter of the arbor (indicated by white line) was defined using the convex hull method. The area covered by the arbor was then divided into ten equally spaced concentric shells (colored white-red) centered on the centroid of the hull. After thresholding, the number of pixels representing axon branches was then quantified for each shell (see Materials and methods). (h) Transverse confocal section through the IPN showing the entire contingent of L and R habenular axon terminals labeled using DiD (red) and DiI (green), respectively, in a Tg(*h2afz*-GFP) transgenic larva to label the nuclei of IPN neurons (blue). Most L-sided neurons innervate the dIPN whereas the majority of R-sided axons terminate in the vIPN. (i, i') L- and R-sided axons labeled in the same larva. L-typical arbors are located dorsal to R-typical arbors. **(j) **Cumulative fraction of axon density plotted against radius (measured from centroid of the convex hull to the perimeter) for ten L-typical and seven R-typical arbors. The data are fit by fourth-order polynomial models (solid lines). Dotted lines indicate the 95% confidence band of each best-fit curve. R-typical arbors have a greater proportion of axon density localized towards the perimeter of the arbor. **(k) **L-typical arbors extend over a greater DV extent than R-typical arbors. **(l) **L-typical arbors have more branch points than R-typical arbors. **(m) **The width/length ratio of L-typical arbors shows a trend towards higher values than for R-typical arbors. **(n) **The majority of L-sided habenular neurons elaborate L-typical arbors whereas most R-sided neurons form R-typical axon arbors. Horizontal lines indicate mean values and error bars show standard error of the mean. ***p < 0.001.

The bilateral projection pattern of individual habenular neurons raises the possibility that axons may form synaptic contacts on both L and R sides of the IPN. Although the IPN is usually described as an unpaired midline nucleus, confocal imaging clearly shows a LR subdivision (Figure 3h). To determine if individual axons are likely to innervate both L and R sides of the IPN, we expressed, by focal electroporation in individual neurons, a construct driving expression of both cytoplasmic red fluorescent protein (RFP) and a Synaptophysin-GFP (Syp-GFP) fusion protein that localizes to presynaptic terminals [17].

Punctate expression of Syp-GFP co-localized with both ipsilateral and contralateral axonal variscosities, strongly suggesting these puncta represent *en passant *pre-synaptic terminals (Figure 2h). Axons from both the L and R habenula appear to form large numbers of synapses on both sides of the midline. We did not observe any obvious differences in the distribution of Syp-GFP puncta between L- and R-sided neurons.

In summary, all of the habenulo-interpeduncular projection neurons that we labeled, both from L and R sides, elaborate remarkable arbors that multiply decussate and establish synaptic contacts on both sides of the brain. Therefore, both L- and R-sided habenular neurons share many features both at the level of soma, dendrite, axon and terminal arbor morphology.

### Habenular projection neurons display one of two discrete sub-types of terminal arbors

Bulk labeling of habenular efferent axons with lipophilic dyes has shown that L and R nuclei differentially innervate dorsal and ventral regions of the IPN [16]. However, the labeling techniques to date have been unable to resolve how this LR asymmetry is reflected at the level of individual neurons. For instance, most data are consistent with two hypotheses: first, individual neurons have terminals that exclusively innervate either the dorsal IPN (dIPN) or the vIPN (and there are different proportions of different classes of neuron on L and R); or second, individual neurons elaborate complex arbors with varying amounts of terminal branches in dorsal versus ventral regions of the target. To resolve this issue, we performed detailed morphometric analyses of terminals from R- and L-sided neurons, focusing on differences that could underlie the lateralization of the circuit.

Terminal arbors of habenular projection neurons adopt one of two very distinct morphologies, which we term L-typical and R-typical (Figure 3; Additional files 2 and 3). Although both types of terminal are exhibited by L and R neurons, they are present at very different frequencies (Figure 3n), with the vast majority (83.8%) of L habenular neurons elaborating L-typical terminal arbors (n = 31/37; Figure 3a–c' and Additional file 2) and 90.5% of R-sided neurons forming R-typical arbors (n = 38/42; Figure 3d–f' and Additional file 3). Three-dimensional reconstructions of L-typical arbors reveal them to be formed like a 'domed crown' with branches extending over considerable DV depth (31.0 ± 1.3 μm). In striking contrast, R-typical arbors are significantly more flattened, extending over less depth (17.7 ± 1.2 μm; *p *< 0.001; Figure 3k).

L-typical arbors have a circular perimeter, surrounding the central 'core' of the IPN. They possess large numbers of branches directed dorsally and medially to form the domed crown of the arbor (Figure 3a–c'). In contrast, R-typical arbors appear more elongated along the anterior-posterior axis. Much of the neurite length is concentrated towards the periphery of the arbor with relatively few branches extending towards the center of the IPN (Figure 3d–f'). Supporting these visual impressions, L-typical arbors display a significantly greater average number of branch points than R-typical arbors (L-typical = 19.3 ± 1.9; R-typical = 7.6 ± 1.2; *p *< 0.001; Figure 3l). In addition, the width/length ratio of L-typical arbors showed a trend towards being greater than that for R-typical arbors, although this difference did not quite reach statistical significance at the 95% confidence level (L-typical = 1.28 ± 0.039; R-typical = 1.13 ± 0.070; *p *= 0.06; Figure 3m).

Finally, we developed a method similar to Sholl analysis to quantify the distribution of neurite branches from the center to the periphery of the arbors (Figure 3g; see Materials and methods). This revealed distinct distribution profiles for L-typical and R-typical arbors (Figure 3j). For L-typical arbors, there is a greater proportion of axon density towards the center of the arbor as a result of the many branches that extend dorsally and medially. By contrast, in R-typical arbors, over 50% of axon density is concentrated within the outer 20% of the arbor radius because more of the axonal length is confined towards the perimeter. Both distributions showed excellent fits to fourth-order polynomial models (L-typical: R^2 ^= 0.9580; R-typical: R^2 ^= 0.9768; 95% confidence intervals for all parameters fit by non-linear regression were small; Additional file 4) and analysis of the curves using Akaike's Information Criterion (AIC) showed they can be considered distinct with greater than 99% probability (ΔAICc = 80.39 for global versus individual fits).

In two cases (from a total of 79 L and R neurons), axon arbors had an intermediate or 'hybrid' morphology (for example, Figure 2g). Of the six L habenular neurons that had R-typical arbors, three had their somata located very close to the midline and actually sent their axons across the dorsal midline of the epithalamus to enter the right FR. Similarly, one of the two R habenular neurons that elaborated a L-typical arbor was located at the most medial edge of the R habenula and extended its axon down the left FR (data not shown).

In summary, although many features are shared by all habenular projection neurons, there are two major sub-types of terminal arbor morphology, one dominant for L-sided neurons and the other for R-sided neurons.

### L-typical and R-typical arbors are elaborated at different DV positions of the target IPN

Do the distinct L-typical and R-typical morphologies arise from axons terminating in different regions of the target, and if so, does this shed light upon the differential innervation patterns of the IPN by L and R habenulae? To answer these questions, we performed anti-GFP immunostaining followed by histological sectioning of the brains of electroporated larvae. We found that L-typical arbors are localized to the dorsal IPN, whereas R-typical arbors are found in the ventral IPN (Additional file 5). In addition, contemporaneous labeling of L and R neurons confirmed that L-typical arbors are located dorsal to the flattened R-typical arbors (Figure 3i,i'). These data indicate that individual habenular neurons innervate either the dIPN or the vIPN but not both domains.

The predominance of ventrally located R-typical terminals on R-sided habenular neurons provides an explanation of why bulk labeling of R habenular efferents leads to predominant labeling of the vIPN [15,16]. The literature is less clear with respect to the efferent connectivity of the L habenula as Gamse and colleagues [15] suggest widespread innervation of the entire IPN by L habenular neurons whereas Aizawa and colleagues [16] propose that the L habenula predominantly innervates the dIPN whilst projecting substantially fewer axons to the ventral target. The predominance of dorsally localized L-typical arbors on L projection neurons supports this latter hypothesis (Figure 3n). However, there remains a small possibility that our electroporation approach is biased to labeling dorsally projecting L habenular neurons. We therefore used lipophilic dyes to label all L- and R-sided efferents in a transgenic line in which dorsal and ventral IPN cells could be visualized.

A transverse confocal view through the IPN of a specimen in which the full contingent of L and R habenular axons have been labeled with different colored lipophilic dyes confirms that the larval L habenula projects predominantly to the dorsal target (Figure 3h). Such images clearly show the dorsal arborization territory in which L-typical arbors form, surrounding and covering the dIPN. In the ventral arborization domain, R-typical arbors surround the vIPN, in a manner reminiscent of an electromagnetic coil.

In summary, the vast majority of L-sided habenular projection neurons form L-typical terminals, restricted to the dIPN, whereas the vast majority of R-sided habenular projection neurons form R-typical terminals in the vIPN.

### Post-synaptic IPN neurons have diverse morphologies

The unique morphologies and DV segregation of habenular axon terminals raises several questions regarding the organization of the target and the morphology of its post-synaptic neurons. For example, do IPN neurons, located in the central 'core', radiate dendritic arbors outwards to synapse with the surrounding afferent axons? What are the consequences of the DV segregation of L-typical and R-typical inputs into the IPN? Are there IPN neurons that exclusively receive inputs from only dorsal or ventral axons or do some neurons synapse with both dorsal and ventral habenular axons, suggesting an integration of predominantly-left and predominantly-right information?

To address these and related questions, we used focal electroporation to label individual IPN neurons with membrane-localized Cherry fluorescent protein in Tg(ET16:GFP) transgenic larvae in which many habenular axons are labeled by GFP expression (the extent of habenular expression is shown in Figure 2c). This allowed us to examine the detailed morphology of IPN neurons whilst localizing their somata and neurite arbors with respect to the dorsal and ventral interpeduncular neuropils.

In total we labeled 20 individual neurons from 14 larvae (4–6 days post-fertilization (dpf)). The vast majority (18/20) were located within the central core of the IPN. Two neurons were located outside, but in very close apposition to, the arborization domains that surround the core. As these neurons extend neurite arbors within the IPN neuropil, we consider them to be interpeduncular neurons.

**Figure 4:**
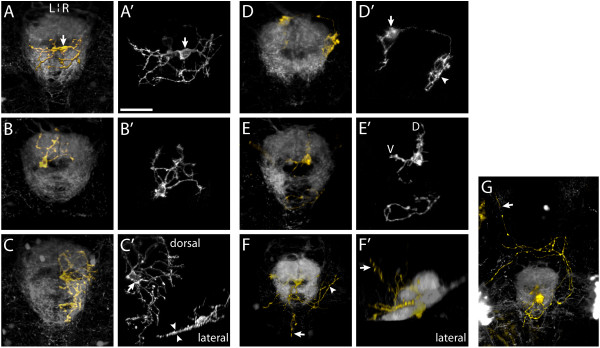
Morphology of IPN neurons. **(a-g, f') **Three-dimensional projections of confocal *z*-stacks showing electroporated IPN neurons expressing membrane-Cherry (gold) and the surrounding IPN neuropil (grey), visualized using the Tg(ET16:GFP) transgenic line. All panels are dorsal views, anterior top, except (f'), which is a lateral view, anterior right. **(a'-e') **Maximum intensity projections of deconvolved confocal *z*-stacks reveal the detailed morphology of IPN neurons. (a, a') An interneuron in the vIPN that extends neurites exclusively within the vIPN neuropil. The soma (arrow) is located on the R of the midline but the neurites enter the neuropil on both L and R sides. A second, more weakly labeled neuron is also visible. (b, b') The soma of this neuron, as with most IPN neurons, occupies the central cellular 'core' of the nucleus whilst its arbor extends radially to synapse with afferent habenular axons that encircle the core. (c, c') A vIPN neuron with a flattened dendritic arbor. In this specimen two neurons were labeled. One of these (soma marked by arrow in (c')) radiates an extensive dendritic arbor exclusively on the R of the midline within the vIPN neuropil. The lateral projection (inset in (c')) reveals the arbor is extremely flattened along the DV axis (arrowheads in (c')). (d, d') Neuron with a dorsally located soma (just outside the dIPN neuropil), which elaborates two arbors in spatially distinct regions of the IPN. The larger of these arbors (arrowhead in (d')) is connected to the cell body (arrow in (d')) by a long, unbranched process. As is also the case for some of the other interneurons, it is unclear whether these arbors are axonal, dendritic or both in nature. (e, e') A dIPN projection neuron that extends an axon just caudal to the IPN, most probably synapsing with neurons of the serotonergic raphé nucleus. This neuron has dendritic processes located both in the dorsal (D) and ventral (V) neuropils. (f, f') In this specimen, three projection neurons are labeled. Axons extend around the IPN (arrowhead in (f)) as well as caudally and dorsally (arrows in (f, f')). **(g) **Projection neurons extending anteriorly directed axons that pass around the IPN, cross the midline and continue rostrally (arrow). Scale bar in (a'): 20 μm

One feature shared by all of the labeled IPN neurons is that they elaborate neurite arbors within the arborization domains of habenular axon terminals that surround and cover the IPN. For IPN neurons with cell bodies within the central core, this means the neurons are polarized such that their neurites extend radially, from the core towards the periphery of the nucleus, to synapse with the surrounding afferent axons (Figure 4a–c).

We classified the IPN neurons as projection neurons (45%, 9/20), if we could observe an axonal projection extending outside the IPN, or as interneurons (55%, 11/20), if the neurites were confined to the IPN. Both interneurons and projection neurons either extended arbors that were restricted to the dorsal *or *ventral neuropil (9/11 interneurons (Figure 4a,a'); 5/9 projection neurons) or innervated both dorsal *and *ventral neuropils (2/11 interneurons; 4/9 projection neurons (Figure 4e,e')). These results show that the IPN contains first, neurons with DV restricted arbors that could specifically relay L-typical or R-typical habenular inputs to downstream target nuclei, and second, neurons that extend neurites into both dorsal and ventral neuropils, which consequently have the potential to integrate predominantly-left and predominantly-right information.

R-typical axon arbors, localized in the vIPN, are often remarkably flattened along the DV axis, having DV extents of as little as 6 μm or less. In two cases we labeled vIPN neurons that also displayed remarkably flattened dendritic arbors (Figure 4c,c'). This suggests that some habenular axon terminals might be topographically arranged within the IPN neuropil and that there might be a precise connectivity between specific pre-synaptic habenular neurons and post-synaptic IPN neurons.

IPN neurons display both 'continuous' and 'split' arbors: The interneurons we labeled, with somata located in the vIPN, typically extended continuous arbors, which varied in size (Figure 4a,a'). By contrast, two dorsal interneurons, and one dorsal projection neuron each elaborated two quite separate arbors that were discretely localized in distinct subdomains of the IPN neuropil (Figure 4d,d').

Projection neurons extended efferent axons to a variety of targets, compatible with reports in other species that the IPN is an integrative center that establishes ascending and descending efferent connectivity with many CNS nuclei [18]. It was common to observe axon terminals in a midline position just caudal to the IPN (Figure 4e,e'). This is the location of the serotonergic raphé nucleus, which is also innervated by the subset of habenular axons that pass around the IPN before converging to the midline (Additional file 1). IPN projection neurons also extended axons caudally towards other sites in the hindbrain as well as to regions of the tegmentum surrounding the IPN (Figure 4f,f',g).

### The parapineal is necessary for laterotopic innervation of the IPN

Previous work has suggested that the parapineal influences asymmetric development of the epithalamus [12,14] and the termination of L habenular efferent axons in the dIPN [15,19] but the proposed re-organization of terminal arbors following parapineal ablation has not been confirmed by definitive axonal tracing experiments.

To explore if and how the parapineal influences the laterotopic targeting of habenular axons and morphology of individual axon terminals, we examined IPN innervation in embryos lacking a parapineal. Using Tg(*flh*:eGFP); Tg(*foxD3*:GFP) transgenic embryos, in which the pineal complex is labeled by GFP expression, we removed parapineal precursors by laser ablation at 24–28 hpf as they start to condense at the midline prior to migration towards the L epithalamus [12]. Successful ablation of all parapineal cells was verified by confocal microscopy at 3 dpf and we subsequently examined various habenular markers or used lipophilic dye tracing to examine habenular connectivity at 4 dpf.

Consistent with previous data [12,14,15], ablation of the parapineal affected gene expression and neuropil organization in the habenulae (Additional files 6 and 7). L-sided *lov *expression, which is normally stronger than in the R habenula, was always reduced, though a small medial domain of expression consistently retained asymmetry (n = 20; Additional file 6b), in a similar region to where a small tuft of medial neuropil labeling also showed asymmetry (Additional file 6f). Complementing the reduction in *lov *expression, L-sided *tag1*, *ron *and *dex *expression were expanded to levels similar to those seen on the R (Additional files 6d and 7, and data not shown). Together, these results show that although parapineal ablation causes a substantial reduction in the magnitude of habenular asymmetry, the subtle LR differences in *lov *expression and neuropil organization suggest that the habenulae might retain distinct characteristics. We next assessed the consequences of parapineal ablation on habenular efferent axons.

**Figure 5:**
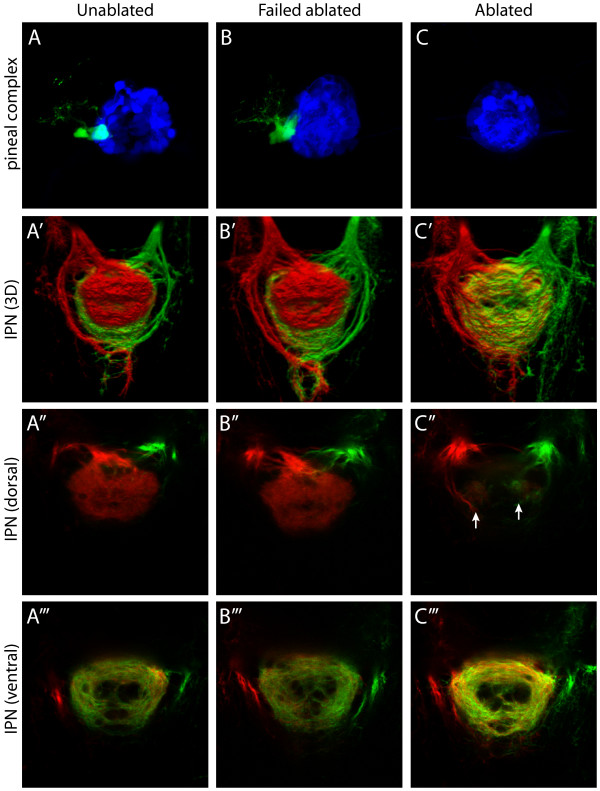
Parapineal ablation eliminates laterotopic habenular efferent connectivity. **(a-c) **Dorsal views of the pineal complex at 3 dpf, visualized in Tg(*flh*:eGFP); Tg(*foxD3*:GFP) transgenic larvae, following laser ablation of parapineal precursor cells at 24–28 hpf. (a) Unablated control larva. (b) Failed ablation control larva, in which the parapineal has not been eliminated. (c) Parapineal-ablated larva, which lacks all parapineal cells. For clarity, pineal cells are pseudocolored blue and parapineal cells pseudocolored green. **(a'-c') **Three-dimensional reconstructions of habenular axon terminals in the IPN following lipophilic dye labeling of L (red) and R (green) habenular neurons. (a", b", c") Single-depth confocal images through the dorsal part of the IPN. (a"', b"', c"') Single-depth confocal images through the ventral part of the IPN. Following parapineal ablation there is an almost complete loss of L habenular innervation of the dIPN (c"). However, two small tufts of neuropil, containing both L- and R-sided axons, are consistently observed in the rostral dIPN (arrows in (c")). In parapineal-ablated larvae there is an increase in the density of L-sided axon terminals in the vIPN (compare (c"') to (a"') and (b"')). All panels show dorsal views, anterior top.

Analyses of IPN innervation using lipophilic tracer dyes to explicity and differentially label L- and R-sided axons in larvae lacking a parapineal clearly demonstrates that this structure influences the ability of L habenular projections to innervate the dIPN. Thus, consistent with our previous data [16], in unablated control larvae (n = 6) and in specimens in which the ablation procedure failed to remove all parapineal precursors (n = 9), L habenular axons show strong innervation of the dIPN and lesser innervation of the ventral target whereas R habenular axons almost exclusively innervate the vIPN (Figure 5a–a"' and 5b–b"'). In parapineal-ablated larvae there is a striking reduction in innervation of the dIPN by L habenular neurons (n = 13; Figure 5c–c"'). Moreover, we consistently observed denser innervation of the vIPN by L-sided axons, suggesting that L habenular projection neurons have re-routed to the ventral target (compare Figure 5a"' or 5b"' with 5c"'). In parapineal-ablated specimens, we also observed two tufts of neuropil at the rostral end of the dorsal nucleus containing both L and, to a lesser extent, R-sided axons (Figure 5c"). Our later experiments provide an explanation for these ectopic projections (see below).

These results confirm and expand upon previous interpretations [15,19] and indicate that the targeting of L habenular axons to the dIPN is almost completely inhibited following parapineal ablation; at the level of analysis feasible with lipophilic dye tracing, the L and R habenulae show similar, symmetric patterns of efferent connectivity.

### In the absence of signaling from the parapineal, L- and R-sided habenular axons retain distinct, lateralized morphologies

Although lipophilic dye tracing confirms that L habenular axons change their projection patterns following parapineal ablation, it does not reveal the underlying alterations in terminal arbor morphology responsible for this change. Two possibilities are either that L-sided axons adopt R-typical morphologies and projection patterns in the absence of the parapineal or that L-sided axons terminate in the same vIPN region as R-sided axons but continue to form arbors with a distinct, lateralized morphology. To address this issue we used focal electroporation to label individual L- and R-sided habenular projection neurons in parapineal-ablated larvae and conducted morphometric analyses of their axonal arbors.

**Figure 6:**
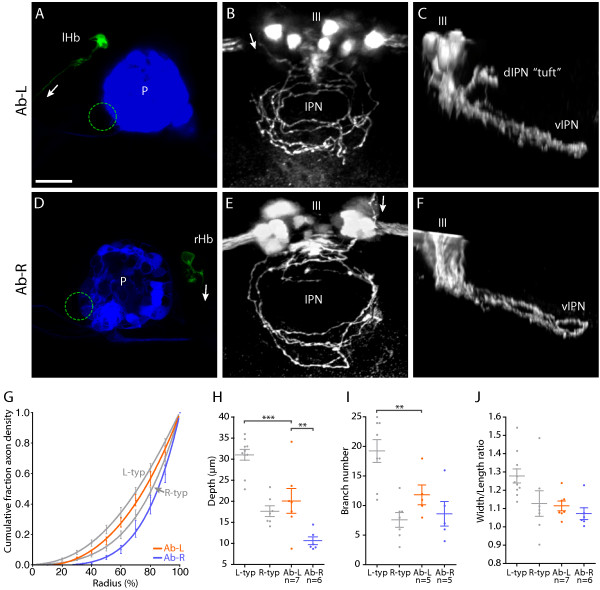
Left and right-sided habenular axons retain distinct morphologies in parapineal-ablated larvae. **(a-f) **Images from brains of 4 dpf Tg(*flh*:eGFP); Tg(*foxD3*:GFP) transgenic larvae in which parapineal ablation was performed at 24–28 hpf and single neurons in the L or R habenula were labeled by focal electroporation at 3 dpf. (a, d) Confocal *z*-projections of the dorsal diencepahlon confirming successful ablation of the parapineal (indicated by dotted circles) and labeling of single L (a) or R (d) habenular (Hb) projection neurons. 'P' indicates pineal. (b, c) Dorsal (b) and lateral (c) views of flattened axonal arbors elaborated by single L-sided neurons in the vIPN after parapineal ablation. Axon branches frequently extend towards the center of the vIPN in these arbors. Some L-sided axons extend collateral branches into the anterior dIPN that terminate with a unique tuft morphology (tuft in (c)). The oculomotor nucleus (III), which lies just anterior to the IPN, expresses GFP in these transgenic larvae and allows the DV position of the arbors to be determined. (e, f) Dorsal (e) and lateral (f) views of arbors formed by single R-sided neurons after parapineal ablation. These arbors appear as a more exaggerated form of the R-typical morphology. Axon branches are strongly localized to the perimeter of the arbor and extend over a very limited DV depth. Arrows indicate the direction of projection of habenular axons. Scale bar in (a): 20 μm. **(g) **Radial distribution of axon density for six Ab-L and five Ab-R arbors. Distribution profiles for L-typical (L-typ) and R-typical (R-typ) arbors are shown in grey. **(h) **Both Ab-L and Ab-R arbors extend over a limited DV depth, similar to R-typical arbors; however, Ab-R arbors are significantly flatter than Ab-L arbors. **(i) **Ab-L arbors have a reduced number of branch points compared to L-typical arbors. **(j) **The width/length ratio of Ab-L and Ab-R arbors are similar to those of R-typical arbors. In (g-j) the L-typical and R-typical data, shown in grey, are the same as presented in Figure 3. Horizontal lines indicate mean values and error bars show standard error of the mean. **p < 0.01; ***p < 0.001.

Following parapineal ablation, L-sided neurons elaborate arbors with a unique 'Ab-L' morphology (for example, Figure 6b,c). By comparing the location of such arbors to that of the oculomotor nucleus, we localized them to the vIPN. This was confirmed by anti-GFP immunostaining followed by histological sectioning (Additional file 5c) and is in agreement with lipophilic dye tracing results (above). These Ab-L arbors extend over a restricted DV depth (20.1 ± 2.9 μm), which is similar to that of R-typical arbors (17.7 ± 1.2 μm) and significantly smaller than for L-typical axons (31.0 ± 1.3 μm) (*p *> 0.05 for R-typical versus Ab-L; *p *< 0.001 for L-typical versus Ab-L; Figure 6h). In addition, in parapineal-ablated larvae, L-sided axons elaborate arbors with significantly fewer branch points (11.8 ± 1.7) than wild-type L-typical arbors (19.3 ± 1.9) (*p *< 0.05 for L-typical versus Ab-L; *p *> 0.05 for R-typical versus Ab-L; Figure 6i) and the overall width/length ratio of Ab-L arbors (1.12 ± 0.03) is similar to that of R-typical axons (1.13 ± 0.07; Figure 6j).

Despite the fact that Ab-L axons project ventrally and display several morphological features characteristic of R-typical arbors, they are not identical to the R-typical arbors of wild-type embryos. Although in Ab-L arbors, many of the terminal neurites surround the central core of the vIPN, they are not concentrated towards the periphery of the arbor to the same extent as we observed for R-typical arbors and, in many cases, the terminal projections of Ab-L axons extend towards the interior of the target nucleus (Figure 6b). The distribution profile of axon density from the center to the perimeter of the Ab-L arbors is intermediate between the more centralized L-typical profile and the more peripheralized R-typical profile (Figure 6g). Moreover, analysis using AIC revealed that the Ab-L profile can be considered distinct from both L-typical and R-typical profiles (L-typical versus Ab-L: ΔAICc = 12.92, 99.84% probability that individual fits are correct versus global fit; R-typical versus Ab-L: ΔAICc = 19.52, >99% probability that individual fits are correct versus global fit).

Although, to date, no changes in differentiation have been described for the R habenula following parapineal ablation, we find that the morphology of R habenular arbors is altered. R-sided neurons in parapineal-ablated embryos elaborate arbors with a more exaggerated form of the wild-type R-typical morphology (for example, Figure 6e). 'Ab-R' arbors are extremely flat (10.6 ± 0.9 μm; *p *< 0.01 for Ab-L versus Ab-R) and their processes are tightly confined to the perimeter of the arbor such that the distribution profile of Ab-R axon density is distinct not only from Ab-L arbors but also from wild-type R-typical arbors (Ab-L versus Ab-R: ΔAICc = 57.5; R-typical versus Ab-R: ΔAICc = 21.55; for both comparisons there is >99% probability that individual fits are correct versus global fit).

In summary, our analyses reveal that in the absence of signaling from the unilateral parapineal, both L and R habenular neurons innervate the vIPN but asymmetry is retained at the level of the morphology and targeting of individual L- and R-sided axon terminals.

### Habenular axons form ectopic terminal tufts into the rostral dIPN in the absence of the parapineal

In parapineal-ablated specimens, we occasionally observed L or R habenular neurons with unusual 'tufts' of processes that projected into the dIPN (3/20 Ab-L axons; 3/32 Ab-R axons; Figure 6c). The arbors comprise highly branched domains on either or both sides of the midline, confined to the anteriormost region of the dIPN. Such tufting of the arbors was never observed for either L- or R-sided habenular neurons in wild-type larvae. These arbors are likely to constitute the small domain of neuropil density in the anterior dorsal IPN when the entire contingent of L- and R-sided axons in parapineal-ablated larvae is labeled by lipophilic dye tracing (see above; Figure 5c").

## Discussion

In this study, we optimized a focal electroporation method that enabled us to resolve how lateralization of the habenulo-interpeduncular circuit is encoded at the level of single neurons. Four principal findings resulted from this. First, all habenular neurons, on both L and R sides, display a stereotypical unipolar morphology and their axons cross the ventral midline multiple times to establish bilateral connectivity. Second, two very distinct axon arbor morphologies are apparent, having lateralized origins and differential target connectivity; this underlies the laterotopic efferent connectivity of the habenulae. Third, IPN neurons display diverse morphologies that suggest lateralized inputs may either be integrated or maintained as distinct circuits, and are relayed to diverse downstream nuclei. Fourth, the unilateral parapineal is essential for the development of both L *and *R habenular axon terminals with appropriate morphology and connectivity; however, the parapineal is not a binary determinant of LR identity, indicating that additional developmental mechanisms are involved in the lateralization of this circuit.

### Habenular projection neurons have a unipolar morphology, and display multiple midline crossing and bilateral connectivity

Despite the fact that the L and R habenulae show conspicuous asymmetries in gene expression, neuropil organization and efferent connectivity, all of the cells we labeled in both the L and R habenulae were projection neurons that shared a stereotypical unipolar morphology. The dendritic arbors of these neurons, located in the central neuropil core of the habenulae, have the potential to integrate asymmetric afferent inputs from the telencephalon and diencephalon [20] and exclusively on the L side, inputs from parapineal axons. Although dye tracing shows the entire contingent of L and R habenular axons terminating on both sides of the midline, our single cell analyses allowed us to establish that individual neurons project to, and establish synaptic contacts on, both L and R sides of the IPN. The repeated recrossing of the midline is a feature perhaps unique to habenular axons, which was previously suggested from classical neuroanatomical studies in mouse and salamander [21,22]. Although in a few rare cases axons can cross from one side of the brain to the other and back again in two separate commissures (for example, [23]), we know of no other examples in normal development of decussating axons that can recross the same midline structure. Indeed, there are evolutionarily conserved mechanisms present from flies to humans by which growth cones become repelled by midline tissue once they have crossed it [24].

A distinctive feature of the habenular axons in zebrafish is that they not only reverse direction from one side of the brain to the other but must also change orientation along the anterio-posterior (AP) axis as they encircle the core of the IPN. This circular spiraling is reminiscent of the aberrant axon pathfinding observed in the ventral nerve cord of *Drosophila *embryos mutant for components of Robo-Slit signaling [25,26].

### Two arbor sub-types with left-right asymmetric origins and distinct target connectivity

Our results reveal how lateralization of the vertebrate CNS is manifest at the level of single axon morphology and connectivity. Despite sharing some morphological characteristics, habenular projection neurons possess one of two strikingly different axon arbor morphologies. L-typical arbors are tall, highly branched and take the form of a 'domed crown'. By contrast, R-typical arbors are often remarkably flattened, with neurite branches concentrated towards the arbor periphery. Although both types of arbor are elaborated by both L- and R-sided neurons, they show strongly lateralized origins, with the vast majority of L-sided neurons having L-typical arbors and most R-sided neurons having R-typical terminals. Because we have found the two arbor sub-types are restricted to different regions of the IPN, their asymmetric origins account for the laterotopic asymmetry in efferent habenular connectivity first identified using lipophilic dye tracing [16].

Although recent studies have shown a temporal bias in the production of neurons in the L and R habenulae [27], we do not think that any of the differences we describe between L-typical and R-typical arbors can be explained by differences in arbor maturity. For instance, we limited our analysis to arbors that had completely encircled the IPN and did not bear visible growth cones on axon branches. In addition, normal L-typical and R-typical morphologies are still present at 10 dpf (data not shown), which suggests the arbors we compared are representative of mature larval morphologies.

Adult L and R habenulae contain medial and lateral subnuclei [15,16], but these sub-divisions are not easily delineated at larval stages (either molecularly or morphologically) and our analysis did not enable us to accurately localize the somata of electroporated neurons to one subnucleus or the other. However, imaging of the epithalamus from the dorsal aspect showed that the neurons we labeled were distributed across the mediolateral extent of both L and R habenulae, suggesting that there is not a significant bias in our sample of L- and R-sided neurons. Although sub-nuclear organization was difficult to assess, one clear correlation between soma location and arbor morphology was that neurons elaborating the minority type of axon terminal arbor (that is, L-sided neurons forming R-typical arbors or R-sided neurons forming L-typical arbors) were often located at the most medial edge of the habenula and extended their axons in the contralateral FR. The relative proportions of L-typical and R-typical neurons we observed on L and R show a good qualitative agreement with subnuclear size ratios as determined by gene/transgene expression and with the strong lateralization evidenced from the laterotopic innervation of the IPN as assessed by lipophilic dye tracing [16].

### The micro-architecture of lateralized neural circuitry

It is currently unknown how lateralization of cognitive function is reflected at the level of circuit micro-architecture. However, several hypotheses could be considered. First, completely unique neuron types and/or circuitry patterns might be specified on L and R. Second, equivalent regions on L and R might both contain the same classes of neuron and patterns of connectivity, but the relative proportions of different neuron types/connections could differ. If this is the case then overall circuit architectures will be distinct, although no unique components need necessarily exist on either side. Third, L and R nuclei might be identical in composition and differ only in size. Cognitive function may then be lateralized as a consequence of more neural substrate existing on L or R. Within the telencephalon-habenula-IPN pathway, there is evidence supporting the first two models. In support of unique circuitry patterns on L and R, the parapineal exclusively innervates the L habenula [12], and a subset of R and L pallial axons terminate exclusively in the R habenula [20].

Our data, together with previous work on habenula-IPN connections [16,27], supports the idea that different proportions of neurons of distinct sub-types contribute to LR differences in circuitry. Thus, we find both L and R habenulae contain neurons with R-typical and L-typical terminals, but in very different proportions. Such a mechanism allows for flexibility and provides an easy way for evolutionary (or indeed developmental) processes to modulate the degree of lateralization by adjusting the difference in ratios of different neuronal types/connections on the two sides. Whilst epithalamic asymmetries are conspicuous in zebrafish and other anamniotes, only subtle LR differences have been described in higher vertebrates [11]. However, it is still possible that, in these species, the habenulae contain distinct classes of projection neuron with different axon terminal morphologies and connectivity preferences, but that the circuit is not strongly lateralized because the proportions of these different neuron types are similar in L and R nuclei. Although functional CNS lateralization is manifest in the form of asymmetric behavioral responses in several species [4], the lateralization of many cognitive functions, such as language processing in the L cerebral cortex, does not result in overtly asymmetric behavioral outputs. The projection of L and R habenular neurons to both sides of the midline IPN potentially provides a mechanism for translating lateralized neural processing into control of bilaterally symmetric downstream circuits. This connectivity pattern enables distinct, asymmetric circuits in the L and R epithalamus to modulate behavioral outputs that require operation of motor circuits on both sides of the animal.

The IPN is a highly conserved structure found in the brains of all vertebrates and has been described as an integrative center connecting limbic regions of the forebrain with hindbrain motor circuits. The IPN is complex with respect to its subnuclear organization and neurotransmitter expression and despite its evolutionary conservation, its specific physiological and behavioral importance is not well understood [18]. Although we find that IPN neurons display diverse morphologies, several characteristics inform hypotheses concerning how lateralized inputs might be integrated or relayed by the IPN.

The fact that L-typical and R-typical arbors are restricted to dIPN and vIPN, respectively, means that post-synaptic IPN neurons with similarly restricted neurite arbors are likely to receive only one sub-type of afferent input. Because the sub-types have strongly lateralized origins, even if the IPN neurons show no selectivity for L- or R-sided axons, they are likely to receive predominantly-left or predominantly-right signals. This suggests the IPN has the capacity to maintain lateralized habenular inputs as largely distinct, independent circuits. A key aim of future studies will be find out if dorsal and ventral IPN projection neurons with DV-restricted dendrites connect to distinct downstream targets.

IPN neurons with neurites in both dorsal and ventral neuropils suggest that the IPN has the capacity to perform a balanced integration of L- and R-sided signals, again without requiring specific recognition of the LR origin of the axons. In this case, lateralized information from the habenulae might be integrated and converge into a common output pathway. Notably, in the IPN of several mammalian species, 'crest' synapses have been described wherein one L and one R habenular axon terminal establish opposing parallel synaptic contacts on either side of a dendritic process belonging to an IPN neuron [28,29], suggesting that the integration of L and R signals is a conserved feature of the IPN.

The strikingly different morphologies of L-typical and R-typical arbors suggest that dorsal and ventral domains of the IPN might process information differently. Within the dorsal IPN, the highly branched, basket-shaped L-typical arbors spread widely over the dIPN cells. This arrangement seems compatible with L-typical neurons providing widespread inputs to this region of the target rather than there being spatially localized, functionally distinct connections. In contradistinction, some R-typical arbors that innervate the vIPN are often remarkably flattened along the DV axis. Correspondingly, we observed that some vIPN neurons radiate similarly flattened, planar dendritic trees. This correlation raises the exciting possibility that the habenulae and vIPN are topographically patterned with different R-typical arbors contacting functionally distinct vIPN neurons.

### The parapineal is necessary for laterotopic habenular connectivity

Our study supports and extends previous observations suggesting that the L-sided parapineal regulates laterotopic connectivity of habenular projection neurons [15,19]. Using lipophilic dye tracing to specifically label L- and R-sided axons in parapineal-ablated larvae, we observed a massive reduction in L-sided innervation of the dIPN and a corresponding increase in innervation of the vIPN. These findings complement a recent study showing that the parapineal is required for expression of a receptor, Nrp1a, which has been proposed to guide L habenular axons to the dIPN [19]. Our data further show that in the absence of the parapineal, the default state is innervation of the ventral target.

### Distinct, lateralized axonal arbors continue to be elaborated by L and R habenular neurons in the absence of the parapineal

Following parapineal ablation, the L habenula adopts patterns of gene expression and neuropil density that are similar to the R habenula (this study and [12,14,15]). However, because subtle asymmetries remain in *lov *expression and neuropil organization (this study and [12]), the extent of the role of the parapineal in specifying LR identity has remained contentious. In this study, we provide clear evidence that although the parapineal regulates aspects of epithalamic asymmetry, L and R habenular neurons remain distinct following parapineal ablation.

Although both L and R habenular axons innervate the vIPN in the absence of the parapineal, they retain distinct terminal arbor morphologies and projection patterns. L-sided neurons form arbors with a unique 'Ab-L' morphology, which, whilst sharing several features of R-typical arbors, show greater branching into the interior of the vIPN. Strikingly, R habenular axons also change their terminal morphology and elaborate arbors that appear as more exaggerated forms of the wild-type R-typical morphology. Because the parapineal is only associated with the L habenula, it seems probable that interactions (direct or indirect) between the L and R axons in the vIPN are responsible for this peripheral restriction of the Ab-R axons: for example, occupancy of the more medial vIPN by Ab-L arbors might displace the Ab-R arbors towards the periphery.

In summary, signaling from the parapineal to L habenular neurons or their precursors is necessary for elaboration of several aspects of the L habenular projection neuron phenotype. These include normal levels of lateralized gene expression and axon targeting to the dIPN. However, even in the absence of the parapineal, L and R axon arbors remain quite distinct, indicating that other factors act upon this circuit to impart LR identity.

### Specification and concordance of LR asymmetries

The various asymmetry phenotypes in the telencephalon-habenula-IPN pathway display a high degree of concordance in wild-type, mutant and experimentally manipulated embryos. The first known step in the development of these concordant asymmetries is the activation of Nodal signaling unilaterally in the L epithalamus during mid-somitogenesis. In fish in which Nodal signaling is either absent or bilaterally symmetric, asymmetries still develop, but their laterality is randomized, suggesting that Nodal is not a determinant of L identity, nor required for asymmetry *per se*, but is required to specify the correct laterality of the asymmetries [13]. Importantly, in these and other experiments it was observed that if one asymmetry phenotype showed reversal (for example, parapineal positioned on the R), this was associated with reversals in other asymmetry phenotypes (for example, habenular neuropil density and *lov *expression). Fish were therefore considered to be either entirely reversed or entirely wild type with respect to these asymmetry characters.

These observations are compatible with the hypothesis that there is a single symmetry-breaking event that results in the L and R sides of the brain being assigned their unique identities. The subsequent development of various L- and R-typical phenotypes would be an invariant and inevitable consequence of the initial event. The unilateral migration of the parapineal was a candidate for such a binary, symmetry-breaking event. Parapineal migration occurs before other overt signs of neuroanatomical asymmetry and in parapineal-ablated larvae the L and R habenulae lose much of their asymmetry. However, as we discuss above, analysis at the level of individual projection neuron morphology has enabled us to determine that asymmetry is retained in the absence of the parapineal. This indicates that either an event earlier than parapineal migration initiates the concordant development of epithalamic asymmetries or that concordance is imposed upon structures that, at least in part, independently initiate the development of asymmetric features.

One obvious possibility is that L-sided Nodal signaling influences L habenular development independent of its influence on parapineal migration and that subsequent interactions between the L habenula and parapineal ensure concordance of laterality in the two structures. In support of this possibility, it is very likely that the Nodal pathway is activated in both parapineal and habenular precursor cells [12]. Furthermore, unpublished results from Myriam Roussigne and Patrick Blader (personal communication) suggest that Nodal signaling may have a direct influence on very early neurogenesis in the L habenula.

In future studies it will be interesting to explore these possibilities. For instance, although technically challenging, it would be informative to know if habenular neurons retain lateralized terminal arbors in fish that lack both a parapineal and lateralized Nodal signaling. It also remains a critical and technically challenging goal to relate the asymmetries in neuroanatomical circuits to behavioral lateralization.

## Conclusion

Focal electroporation has enabled us to examine the organization of lateralized neural circuitry at single-cell resolution. We find that two distinct sub-types of projection neuron – that differ in axonal morphology and connectivity – are specified in both left and right habenulae, but that conspicuous differences in their relative ratios result in the major asymmetry in habenular efferent connectivity. This strategy of utilizing the same circuitry components on both sides but adjusting cell-type compositions so as to produce unique, asymmetric, circuit architectures is likely to account for neural asymmetries in other sites in the CNS in different vertebrate species. Furthermore, the morphologies of single post-synaptic neurons suggest lateralized habenular inputs may either be integrated within the IPN or maintained as largely independent circuits. These analyses of the microarchitecture of asymmetric neural tissue are likely to represent an important step towards understanding the basis for lateralization of neural processing and cognition.

## Materials and methods

### Zebrafish lines

Embryos and larvae were obtained by natural spawning from wild-type, Tg(*fox*D3:GFP) [12,30], Tg(*flh*:eGFP); Tg(*foxD3*:GFP) [12], Tg(*h2afz*-GFP) [31], or Tg(ET16:GFP) fish (a gift from Dr Vladimir Korzh). The ET16 enhancer trap line carries a Tol2-GFP insertion and labels a subset of habenular neurons [32,33]. Embryos were reared and staged according to standard procedures [34] and occasionally 0.002% phenylthiourea was added to the fish water from 24 hpf to inhibit pigment formation.

### Dye labeling

Carbocyanine dye labeling of habenular efferent axons was performed as described previously [16].

### Laser ablation

Laser ablation of parapineal precursors was performed at 24–28 hpf in Tg(*flh*:eGFP); Tg(*foxD3*:GFP) transgenic embryos as described previously [12]. Larvae were subsequently examined by laser-scanning confocal microscopy at 3 or 4 dpf to determine if any parapineal cells remained. Larvae lacking all parapineal cells were classed as 'ablated' whereas those retaining one or more parapineal cell(s) were classed as 'failed ablated'.

### Focal electroporation

The electroporation technique was adapted from [35] to enable the efficient transfer of DNA to single cells or small group of cells in embryonic zebrafish CNS. Embryos at 48–72 hpf were mounted in 2% low melting point agarose (Sigma-Aldrich, St Louis, MO, USA) and using a microsurgical blade, a small chamber of agarose was cut out to expose the dorsal diencephalons/mesencephalon. Micropipettes with a tip diameter of 1–2 μm were pulled on a P-87 micropipette puller (Sutter Instrument Company, CA, USA) using AlSi glass capillaries containing a filament. Micropipettes were filled with a solution containing purified plasmid DNA resuspended in H_2_O at a concentration of 1 μg/μl. For most habenular neuron electroporations we used pCS2-GAP43-GFP (a gift from Dr E Amaya). GFP synthesized from this construct is localized to the cell membrane by virtue of two amino-terminal palmitoylation signals from the GAP43 protein. To visualize presynaptic terminals, we used a 1:1 mixture of pCS2-GAL4 plasmid DNA (a gift from Dr Masahiko Hibi) and pCS2-Syp:GFP-DSR [17]. This latter construct encodes both cytoplasmic DsRed fluorescent protein and a Syp-GFP fusion protein, driven from separate UAS elements. For IPN electroporations we used pCS2-lyn-Cherry, which encodes a membrane-targetted Cherry fluorescent protein (a kind gift from Henry Roehl). Micropipettes were guided into either the L or R habenula or the IPN using an MX3000 Huxley-style micromanipulator (Soma Scientific Instruments, Irvine, CA, USA) under ×40 water-immersion DIC optics (Axioskop 2 FS microscope, Carl Zeiss). The following stimulation parameters were used: 1–2 s long trains of 2 ms square pulses at 200 Hz and a potential difference of 30 V. Trains were delivered 3–5 times with approximately 0.5 s interval between trains. Pulses were generated with a Grass SD9 stimulator (Grass-Telefactor, West Warwick, RI, USA). After electroporation, embryos were cut out from the agarose and returned to embryo medium.

### Whole mount *in situ *hybridization and immunostaining

*In situ *hybridization, antibody staining and histological sectioning were performed according to standard methods [36]. For antibody stainings, mouse anti-acetylated tubulin (T6793; Sigma) and rabbit anti-GFP (TP401; Torrey Pines Biolabs, San Diego, CA, USA) were used at 1:1,000 dilutions and rabbit anti-DsRed (632496; ClonTech, Palo Alta, CA, USA) was used at 1:600.

### Microscopy and image manipulation

Fluorescent labeling was imaged by confocal laser-scanning microscopy (Leica SP2) using ×40 and ×63 water-immersion objective lenses. *z*-stacks were typically acquired at 1–2 μm intervals for epithalamic labeling and fluorescent dye-labeling of habenular axons or 0.5–1 μm intervals for imaging axonal arbors or IPN neurons labeled by electroporation. In some cases, *z*-stacks were deconvolved using Huygen's Essential software (Scientific Volume Imaging, Hilversum, The Netherlands). Three-dimensional projections were generated from the stack of images using Volocity software (Improvision, Coventry, UK).

*In situ *hybridization staining and plastic sections were photographed using a Jentopix C14 digital camera attached to a Nikon Eclipse E1000 compound microscope. For presentation, image manipulation was performed using Photoshop CS2 (Adobe) software.

### Morphometric analyses

#### Radial distribution of neurites

To quantify the distribution of neurite branches from the center to periphery of each terminal arbor, we developed a method similar to Sholl analysis. Three-dimensional reconstructions of each arbor were orientated such that the base of the arbor lay on a flat plane and a two-dimensional image of the reconstruction, parallel to this plane, was used for further analysis. The incoming axon was cropped where it extended beyond the maximum width and length of the arbor. Next, the image was thresholded and the convex hull method was used to define the arbor perimeter (ImageJ software, US National Institutes of Health, Bethesda, Maryland, USA; Hull and Circle plug-in by A Karperien and TR Roy). Using custom-written MATLAB software (The MathWorks, Inc., Natick, MA, USA), a series of 10 equally spaced concentric shells were defined, centered upon the centroid of the convex hull (see Figure 3g, for example). The number of pixels (representing axon signal) in each shell was taken as a measure of axon density. This generated a plot of cumulative fraction of axon density versus radius, for each arbor. This method is resistant to differences in the absolute area covered by the arbor and the total axonal length. Because we analyzed two-dimensional images, our method will underestimate axon density where axon segments are aligned above or below one another. This occurs rarely for L-typical arbors but is more common at the perimeter of R-typical arbors. Thus, although our method detects a greater peripheral localization of axonal length in R-typical arbors, if anything this difference between the arbor sub-types is likely to have been underestimated.

To describe the distribution profiles for the different sub-types of arbor (L-typical, R-typical and Ab-L, Ab-R) non-linear regression was used to fit fourth order polynomial models to the raw data with the y-intersect constrained to zero (at 0% radius the cumulative fraction of axon density must be zero). To compare the curves for the different arbor sub-types, we used the AIC method [37,38]. Briefly, we used the AIC method to compare two models; an AICc score is computed for a 'global' model that treats all the data from two arbor sub-types as a single data set and for a second model with individual curves fit to each data set. A large difference in the AICc scores, ΔAICc, indicates there is a high probability of the model with the lower AICc score being correct. If this is the model with separate fits for the two arbor sub-types it follows that the sub-types can be considered distinct. In the Results text we report ΔAICc and the probability that the 'individual' model, with separate polynomial fits for the two arbor sub-types, is correct.

#### Width/length ratio

The maximum length (measured along the AP axis) and maximum width (measured perpendicular to the AP and DV axes) were measured (Volocity, Improvision). Width/Length ratios were compared using one-way ANOVA with Tukey's post-tests for pair-wise comparisons of arbor sub-types.

#### Depth

The depth over which each axon elaborated its terminal arbor was measured in YZ projections made using Volocity software. For L-typical arbors located in the dIPN, depth was measured parallel to the DV axis of the brain. Because the neuropil domain of the vIPN is inclined relative to the DV axis, accurate depth measurements for ventrally located R-typical, Ab-L and Ab-R arbors were made perpendicular to the plane of the vIPN neuropil domain. Depths were compared using one-way ANOVA with Tukey's post-tests for pair-wise comparisons of arbor sub-types.

#### Branching

The number of branch points was counted by hand in three-dimensional reconstructions of axonal arbors. Branch points giving rise to small filopodial extensions (less than 5 μm in length) were excluded from the analysis. Average numbers of branch points were compared by one-way ANOVA with Tukey's post-tests for pair-wise comparisons of arbor sub-types.

#### Statistics

All statistical comparisons, nonlinear regression and comparison of curves using the AIC method were performed using Prism 4 (GraphPad Software Inc., San Diego, CA, USA).

## Abbreviations

AIC, Akaike's Information Criterion; AP, anterio-posterior; CNS, central nervous system; dIPN, dorsal IPN; dpf, days post-fertilization; DV, dorso-ventral; FR, fasciculus retroflexus; GFP, green fluorescent protein; hpf, hours post-fertilization; IPN, interpeduncular nucleus; L, left; R, right; RFP, red fluorescent protein; Syp-GFP, Synaptophysin-GFP; vIPN, ventral IPN.

## Competing interests

The author(s) declare that they have no competing interests.

## Authors' contributions

IB designed and performed all the experiments, analyzed the data and wrote the manuscript with SW. MC performed *in situ *hybridization assays. CR provided training in laser ablation and assisted with experimental design. JC provided training in focal electroporation and experimental design. SW designed and conceived the study and wrote the manuscript with IB. All authors approved the manuscript.

## Supplementary Material

Additional file 1 A subset of habenular projection neurons extend axons that pass around the IPN and terminate in the anterior hindbrain. **(a, b) **Confocal *z*-projections of the ventral midbrain and anterior hindbrain in 5 dpf (a) and 8 dpf (b) larvae in which groups of habenular neurons have been labeled by focal electroporation. Some habenular neurons project axons that course ipsilaterally around the IPN (arrows) before converging medially to terminate on either side of the midline. These caudal terminations lie in the anterior hindbrain at the level of the serotoninergic raphé nucleus [16]. Scale bar: 25 μm. Click here for file

Additional file 2 Movie of a L-typical axon arbor. Example of a L-typical axon arbor elaborated by a L-sided habenular neuron in the dIPN. Click here for file

Additional file 3 Movie of a R-typical axon arbor. Example of a R-typical axon arbor elaborated by a R-sided habenular neuron in the vIPN. Click here for file

Additional file 4 Non-linear regression. Details of the non-linear regression used to fit fourth-order polynomial models to the radial distribution of axon density data for the different sub-types of axon arbor. Click here for file

Additional file 5 Localization of habenular axon arbors to the dIPN or vIPN. **(a-c) **Transverse plastic sections through the IPN of larvae in which habenular neurons were labeled by focal electroporation, followed by anti-GFP immunostaining (brown) to determine the location of axon arbors (indicated by arrows). (a) L-typical arbors are localized in the neuropil surrounding and covering the dIPN. (b, c) By contrast, R-typical terminals (b) and Ab-L terminals (c) are located in the vIPN. For (c), the parapineal ablation was performed in a Tg(*flh*:eGFP); Tg(*foxD3*:GFP) transgenic embryo in which GFP is weakly expressed in the habenular axons innervating the vIPN. The strongly labeled Ab-L terminals are seen as dark puncta (arrows in (c)) within the more lightly stained vIPN neuropil. Panels show transverse sections through 10 dpf (a, b) or 4 dpf (c) larval brains. Dotted white lines indicate the boundary between the dorsal and ventral parts of the IPN. Click here for file

Additional file 6 Parapineal ablation causes a substantial reduction in epithalamic asymmetries. (a-f) Dorsal views of the epithalamus in larvae in which parapineal ablation was performed at 24–28 hpf, and gene expression and neuropil organization were assessed at 4 dpf. (a, b) In the parapineal-ablated larva, *lov *expression is substantially reduced in the L habenula to levels similar to the R habenula. However, a small, asymmetric, medial expression domain is retained (arrow in (b)). (c, d) *ron *expression appears bilaterally symmetric in the parapineal-ablated larva. (e, f) Anti-acetylated tubulin immunostaining reveals a considerable reduction in the size of the asymmetric dorsomedial neuropil domain in the L habenula after parapineal ablation. However, a small medial 'stump' is retained (arrows). All panels show dorsal views, anterior top. Click here for file

Additional file 7 Summary of effects of parapineal-ablation upon expression of habenular marker genes. Quantification of various habenular markers in parapineal-ablated and control larvae. Click here for file
