# Supplementary figures and images for: Brain asymmetry is encoded at the level of axon terminal morphology
Source: Neural Dev. 2008 Mar 31;3:9. doi: 10.1186/1749-8104-3-9 (PMC2292717; doi:10.1186/1749-8104-3-9)

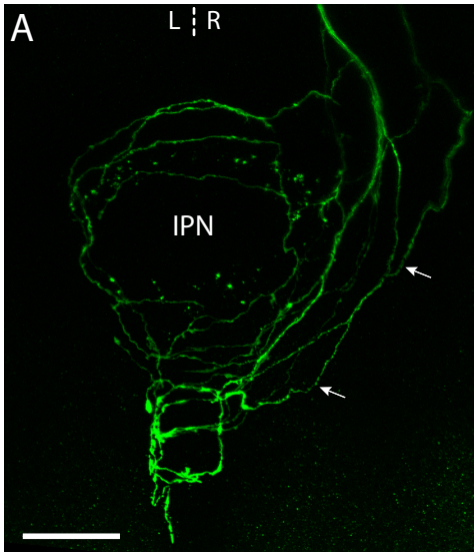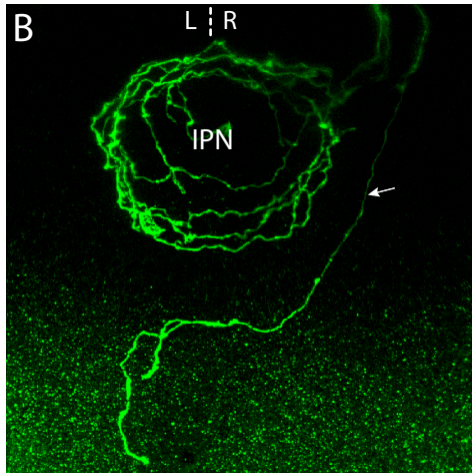

Supplement: Additional file 1 — A subset of habenular projection neurons extend axons that pass around the IPN and terminate in the anterior hindbrain. (a, b) Confocal z-projections of the ventral midbrain and anterior hindbrain in 5 dpf (a) and 8 dpf (b) larvae in which groups of habenular neurons have been labeled by focal electroporation. Some habenular neurons project axons that course ipsilaterally around the IPN (arrows) before converging medially to terminate on either side of the midline. These caudal terminations lie in the anterior hindbrain at the level of the serotoninergic raphé nucleus [16]. Scale bar: 25 μm. [file 1749-8104-3-9-S1.pdf]

L-typ terminals, 10 dpf

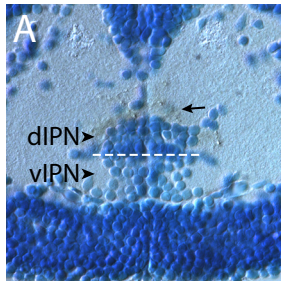

R-typ terminals, 10 dpf

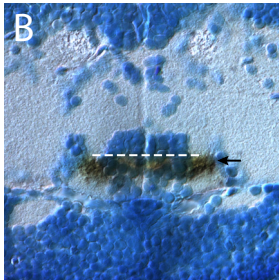

Ab-L terminals, 4 dpf

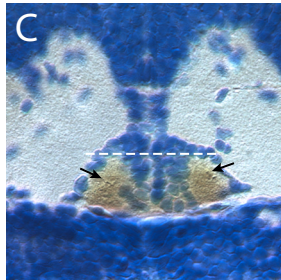

Supplement: Additional file 5 — Localization of habenular axon arbors to the dIPN or vIPN. (a-c) Transverse plastic sections through the IPN of larvae in which habenular neurons were labeled by focal electroporation, followed by anti-GFP immunostaining (brown) to determine the location of axon arbors (indicated by arrows). (a) L-typical arbors are localized in the neuropil surrounding and covering the dIPN. (b, c) By contrast, R-typical terminals (b) and Ab-L terminals (c) are located in the vIPN. For (c), the parapineal ablation was performed in a Tg(flh:eGFP); Tg(foxD3:GFP) transgenic embryo in which GFP is weakly expressed in the habenular axons innervating the vIPN. The strongly labeled Ab-L terminals are seen as dark puncta (arrows in (c)) within the more lightly stained vIPN neuropil. Panels show transverse sections through 10 dpf (a, b) or 4 dpf (c) larval brains. Dotted white lines indicate the boundary between the dorsal and ventral parts of the IPN. [file 1749-8104-3-9-S5.pdf]

Unablated

Ablated

*lov*

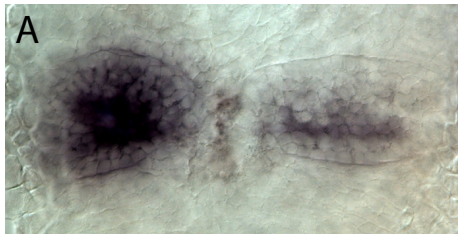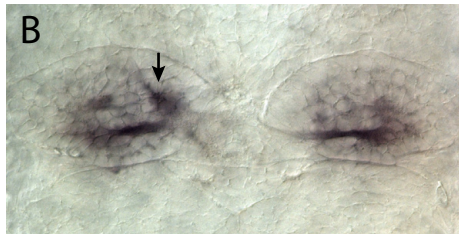

*ron*

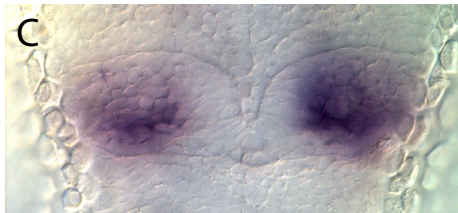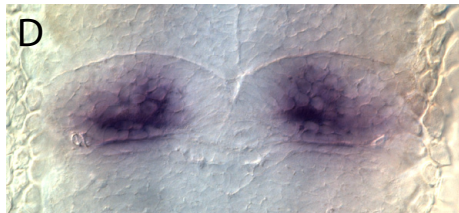

*neuropil*

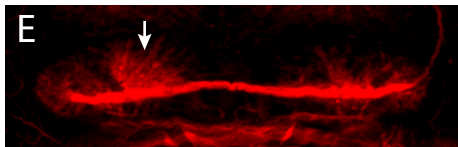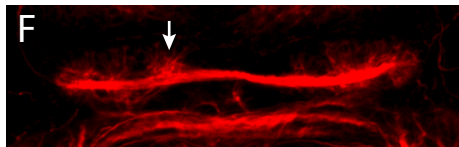

Supplement: Additional file 6 — Parapineal ablation causes a substantial reduction in epithalamic asymmetries. (a-f) Dorsal views of the epithalamus in larvae in which parapineal ablation was performed at 24–28 hpf, and gene expression and neuropil organization were assessed at 4 dpf. (a, b) In the parapineal-ablated larva, lov expression is substantially reduced in the L habenula to levels similar to the R habenula. However, a small, asymmetric, medial expression domain is retained (arrow in (b)). (c, d) ron expression appears bilaterally symmetric in the parapineal-ablated larva. (e, f) Anti-acetylated tubulin immunostaining reveals a considerable reduction in the size of the asymmetric dorsomedial neuropil domain in the L habenula after parapineal ablation. However, a small medial 'stump' is retained (arrows). All panels show dorsal views, anterior top. [file 1749-8104-3-9-S6.pdf]
