# Supplementary material for: Brain asymmetry is encoded at the level of axon terminal morphology
Source: Neural Dev. 2008 Mar 31;3:9. doi: 10.1186/1749-8104-3-9 (PMC2292717; doi:10.1186/1749-8104-3-9)
Supplement: Additional file 4 — Non-linear regression. Details of the non-linear regression used to fit fourth-order polynomial models to the radial distribution of axon density data for the different sub-types of axon arbor. [file 1749-8104-3-9-S4.pdf]

Non-linear regression

	L-typical	R-typical	Ab-L	Ab-R
Best-fit values				
A	0	0	0	0
B	0.0006474	1.44E-06	-0.001104	-0.0007964
C	4.47E-05	3.19E-05	7.12E-05	4.18E-05
D	9.30E-07	-4.47E-08	3.78E-07	-8.66E-07
E	-4.41E-09	7.20E-09	1.08E-10	1.52E-08
Std. Error				
B	0.002312	0.002001	0.00264	0.003289
C	0.0001212	0.0001049	0.0001384	0.0001724
D	1.98E-06	1.71E-06	2.26E-06	2.81E-06
E	1.01E-08	8.72E-09	1.15E-08	1.43E-08
95% Confidence Intervals				
B	-0.003949 to 0.005244	-0.003996 to 0.003999	-0.006395 to 0.004187	-0.007423 to 0.005830
C	-0.0001963 to 0.0002856	-0.0001776 to 0.0002415	-0.0002062 to 0.0003486	-0.0003057 to 0.0003892
D	-3.002e-006 to 4.861e-006	-3.463e-006 to 3.374e-006	-4.147e-006 to 4.903e-006	-6.533e-006 to 4.801e-006
E	-2.445e-008 to 1.563e-008	-1.023e-008 to 2.463e-008	-2.296e-008 to 2.318e-008	-1.373e-008 to 4.405e-008
Goodness of Fit				
Degrees of Freedom	96	66	56	46
**R squared**	**0.958**	**0.9768**	**0.9677**	**0.9571**
Absolute Sum of Squares	0.4659	0.1679	0.2126	0.2259
Sy.x	0.06966	0.05043	0.06161	0.07007
Runs test				
Points above curve	58	36	37	30
Points below curve	42	34	23	20
Number of runs	47	33	25	15
P value (runs test)	0.3236	0.2753	0.1442	0.002218
Deviation from Model	Not Significant	Not Significant	Not Significant	Significant
Constraints				
A	A = 0.0	A = 0.0	A = 0.0	A = 0.0

Radial distribution of axon density data was fit by non-linear regression with a fourth-order polynomial model ($Y = A + B*X + C*X^2 + D*X^3 + E*X^4$). The value of the y-intersect was constrained to zero (A=0) because no axon density can have been accumulated at 0% radius. The polynomial model produced a good fit for all four data sets. R^2 values were high and 95% confidence intervals of all the parameters fit by regression were small.
