# Supplementary material for: Brain asymmetry is encoded at the level of axon terminal morphology
Source: Neural Dev. 2008 Mar 31;3:9. doi: 10.1186/1749-8104-3-9 (PMC2292717; doi:10.1186/1749-8104-3-9)
Supplement: Additional file 7 — Summary of effects of parapineal-ablation upon expression of habenular marker genes. Quantification of various habenular markers in parapineal-ablated and control larvae. [file 1749-8104-3-9-S7.pdf]

Summary of effects of parapineal-ablation upon expression of habenular marker genes

Gene/Marker	Age (dpf)	Phenotype*	Unablated	Failed ablation	Ablated
lov	4	L>R (WT asymm)<br>L>R (medium)<br>L>R (weak)	100%<br>0%<br>0%	100%<br>0%<br>0%	0%<br>15%<br>85%
		n	8	8	20
ron	4	R>L<br>R=L	83%<br>17%	-<br>-	0%<br>100%
		n	6		7
dex	4-5	R>L<br>R=L	90%<br>10%	67%<br>33%	27%<br>73%
		n	20	3	15
Anti-Acetylated tubulin immunostaining (neuropil)	4	L>R<br>L>R (retaining truncated medial tuft)	100%<br>0%	-<br>-	0%<br>100%
		n	7		11

* For *lov* expression, L>R (WT asymm) indicates the wild-type pattern of *lov* expression. L>R (weak) indicates *lov* was expressed at low levels, characteristic of the wild-type right habenula, on both sides but the left habenula showed a small additional domain of medial expression. L>R (medium) clearly showed stronger expression on the left, but not to the same extent as for wild-type specimens.
